# A Universal Spring-Probe System for Reliable Probing of Electrochemical Lab-on-a-Chip Devices

**DOI:** 10.3390/s140100944

**Published:** 2014-01-08

**Authors:** Moon-Keun Lee, Tae Jae Lee, Ho Woon Choi, Su Jeong Shin, Jung Youn Park, Seok Jae Lee

**Affiliations:** 1 Center for Nanobio Integration & Convergence Engineering, National Nanofab Center, 291 Daehak-ro, Yuseong-gu, Daejeon 305-806, Korea; E-Mails: mklee@nnfc.re.kr (M.-K.L.); tjlee@nnfc.re.kr (T.J.L.); hwchoi@nnfc.re.kr (H.W.C.); sjshin@nnfc.re.kr (S.J.S.); 2 National Fisheries Research and Development Institute, 216 Haean-ro, Gijang-up, Gijang-gun, Busan 619-705, Korea; E-Mail: genome@korea.kr

**Keywords:** spring-probe, reliability, probing system, electrochemical measurement, lab-on-a-chip

## Abstract

For achieve sensitivity in lab-on-a-chip electrochemical detection, more reliable probing methods are required, especially for repeated measurements. Spring-probes are a promising candidate method which can replace needle-like probes and alligator clips that usually produce scratches on the surface of gold electrodes due to the strong physical contacts needed for electrochemical measurements. The superior reliability of amperometric measurements by a spring-probe system was compared with results by conventional probing methods. We demonstrated that a universal spring-probe system would be potentially suitable to achieve high performance in lab-on-a-chip devices using electrochemical detection.

## 1. Introduction

Lab-on-a-chip (LOC) technology is widely studied for various applications, with electrochemical reactions being utilized to perform detection, pumping, separation, and capillary electrophoresis [1–4]. Electrochemical detection is one of the most critical processes to determine the performance of a LOC device. Especially in biosensing devices using microfluidic chips, electrochemical detection has been recognized to provide easier detection technology and the wider applicability than the conventional fluorescence technology, because it does not need labeling for target analytes, light sources for excitation and had no transparent material limit for substrates [5]. As a result, various biosensing devices using electrochemical transducers have been developed in the last decade with high sensitivity corresponding to the results achievable using fluorescent labeling methods [6–9].

In addition to the sensitivity, device reliability is another issue to be ensured, even in laboratory-level experiments, for accurate detection with high sensitivity [10]. To obtain higher reliability in electrochemical detection, sample preparation methods and analyte selection have been the focus so far. These components are related to the performance of the transducer which generates the electrochemical detection signal. However, the components of the electrical signal path after the transducer such as conductive metal lines, electrodes and probes have not been investigated, and these components might have been assumed to work ideally without degradation of reliability.

This assumption is based on the metallization of gold for conductive layers as well as the electrodes of the LOC. The electrical properties of gold are excellent enough to remove any concerns about degradation of signal-to-noise. Contrary to its superior electrical properties, the mechanical properties of gold are inferior to those of other metals. The shear modulus and Vickers hardness of gold are 27 GPa and 216 MPa, respectively, which are only about one sixth and one sixteenth, compared to tungsten, one of the hard metals [11]. The poor mechanical characteristics of gold may cause the failure to make a stable electrical contact between the electrode and the probe in a probing system. The electrical contact may become unstable, especially for the detections that require repeated probing on the same gold electrodes. For instance, electrochemical detection of deoxyribonucleic acid (DNA) hybridization usually needs two time measurements, before and after the hybridization process in an oven at an elevated temperature.

Several probing methods have been utilized for the contact on gold electrodes [12–14]. A conductive adhesive such as silver paste may provide stable contact connecting to a potentiostat only for one time probing of electrochemical measurements, but probing with a conductive adhesive is destructive once it is hardened. A needle-like probe or an alligator clip can be a better method for multiple measurements as they work by physical contact. However, there is still the issue of the potential destruction of the gold electrode due to scratching or tearing by shear force when the experiment requires alternating attachment and detachment processes.

In this article, we demonstrate how electrochemical measurement is affected by the deformation of gold electrode and suggest a novel probing method, the universal spring-probe system, which provides repeated multiple stable contacts, enabling a consistent electrochemical detection throughout experiments. The practicality of the universal spring-probe system will be specifically demonstrated in the detection of DNA hybridization that needs two measurements before and after hybridization.

## 2. Experimental Section

### 2.1. Fabrication of the Spring-Probe System

The two parts of the spring-probe system, the top cover and bottom case, were made of cyclic olefin copolymer (COC). The flat COC plate (80 × 80 mm, 5 mm thick) of the top cover was drilled using a computer numerical control machine (TinyCNC-6060C, Tinyrobo, Bucheon, Korea) to make 0.9 mm holes for the spring-probes (G070R, Leeno Industrial Inc., Busan, Korea). The resistance of the spring-probes is less than 50 mΩ. Spring-probes were inserted into the holes and then firmly attached by instant adhesive or fixed by rubber rings. A rectangular (60 × 60 mm, 6 mm deep) crater was processed in another flat COC plate (80 × 80 mm, 10 mm thick) for the bottom case to set the LOC in.

### 2.2. Fabrication of the LOC

The LOC was composed of three COC plates (60 × 60 mm, 1 mm thick) that were formed by an injection mold machine (A270C400-100, Arburg GmbH, Lobburg, Germany). The lower one was a flat COC plate. The middle one had open areas for probing, energy directors for ultrasonic welding and inlet and outlet structures. The upper one also had open areas for inlet, outlet and probing. For electrode patterning, gold (200 nm) with chromium (20 nm) as adhesion layer were deposited on the lower COC plate with shadow mask using an evaporator (EBS400, Evatec, Flums, Switzerland).

Double-side adhesive polyimide film (60 × 60 mm, 0.2 mm thick) was utilized to form the microfluidic channels as well as to bond the lower and middle plates. A specially designed knife was used to cut out the patterns for the microfluidic channels and open area. After removing the liner film, double-side adhesive polyimide film was placed on the middle COC plate and then the lower COC plate was attached to it. This bonding work was carefully performed to align the microfluidic channels to the gold electrodes. Rubbing or pressing was demanded during this work to avoid trapping air in the adhesive interfaces. Finally, the upper COC plate was attached to the adhesively bonded assembly using an ultrasonic welder (2000X F/aef, Branson, Danbury, CT, USA). At this time, rubber caps were inserted between the middle and upper COC plates to provide inlets and outlets where the solution would be injected or drained using a syringe needle.

### 2.3. Electrochemical Measurements Using the Spring-Probe System

Ferricyanide ([Fe(CN)_6_]^3−)^ solution was prepared by mixing in 1:1 proportion potassium hexacyanoferrate (III) (K_3_Fe(CN)_6_, Sigma-Aldrich, St. Louis, MO, USA) solution in deionized (DI) water with potassium hexacyanoferrate (III) trihydrate (K_3_Fe(CN)_6_·3H_2_O, Sigma-Aldrich) solution in DI water. One hundred mM ferricyanide solution was injected as electrochemical mediator into the microfluidic channel without trapping air bubbles.

Working, counter and reference electrodes for electrochemical measurements were made of gold. Five amperometric I-T curves (5 mV, 1 s, 0.01 s interval) were obtained using a potentiostat (760E, CH Instruments Inc., Austin, TX, USA) in the needle-like probing, alligator clipping and spring-probe system configurations, respectively. The needle-like probe was mechanically controlled to land on the gold electrode while the alligator clip was handled manually. In the case of the spring-probe system, the microfluidic LOC was set in the bottom case and then the top cover was vertically enclosed with a vise clip. Each probing method was disassembled after each electrochemical measurement and then reassembled for the next measurement.

### 2.4. Detection of DNA Hybridization

Probe DNA was prepared by synthesis, having a sequence of 5′-thiol(C6)-TTTTTTTTT TATAAAACCCCCAGGCACCAC-3′ for perfect match (PM) and 5′-thiol(C6)-TTTTTTTTTCTCTG TTCGTCTGATCTGTTC-3′ for mismatch (MM). Probe DNA (10 μM) was immobilized on the working electrodes of the lower COC plate using the spotting method for 2 h before bonding to the middle COC plate. After immobilization, DNA-immobilized electrodes were washed out using phosphate buffered saline (PBS, 10 mM, pH 7.4) solution, which was prepared by dissolving PBS tablets (Sigma-Aldrich) in DI water, followed by blocking DNA for 1 h using a solution that was prepared by diluting 1 μL of 6-mercaptohexanol (HS(CH_2_)_6_OH, Sigma-Aldrich) with 5 mL of DI water. Lastly, all working electrodes were fully rinsed using DI water and PBS solution.

Hybridization solution was prepared by mixing in 9:1 proportion 3× saline-sodium citrate (SSC) buffer (Sigma-Aldrich) with 0.3% *N*-lauroylsarcosine sodium salt (Sigma-Aldrich) and target DNA solution that was generated from polymerase chain reaction using genomic DNA of Korean pomfret. For DNA hybridization, the chip was incubated in oven at 57 °C for 1.5 h. The target DNA sequence was TTCTTTATAGTAATGCCAGTTATAATTGGAGGATTTGGTAATTGACTTGTCCCTATAATAATTGGGGCCCCTGACATAGCATTTCCTCGAATGAATAACATAAGCTTTTGACTCTTACCCCCATCTTTCTTACTTCTACTAGCCTCTTCAGGAGTCGAAGCTGGTGCCGGAACCGGATGAACAGTCTACCCACCATTGGCTGGTAACCTTGCCCATGCTGGGGCATCCGTTGACTTAACTATTTTTTCCCTACATTTGGCAGGGGTATCTTCAATTCTCGGAGCTATTAATTTCATTACAACCATCATTAATATAAAACCCCCAGGCACCACCCAATACCAAACACCTCTCTTTGTCTGAGCCGTATTAATTACAGCCGTTCTTCTTCTTTTATCCCTACCAGTTCTTGCTGC.

Two working electrodes were assigned to PM and MM for spring-probing and another two working electrodes to PM and MM for alligator clipping. Five cyclic voltammograms (−0.6 V to 0.6 V, 0.1 V/s, 0.001 s intervals) were obtained at each working electrode using a potentiostat before and after hybridization, respectively. Ferricyanide solution (100 mM) used as electrochemical mediator was injected into the microfluidic channel and washed out using PBS solution after the measurement. Oxidation peaks and reduction peaks were calculated from the cyclic voltammograms to extract the differences in peak current after DNA hybridization.

## 3. Results and Discussion

### 3.1. Universal Spring-Probe System

**Figure 1.**
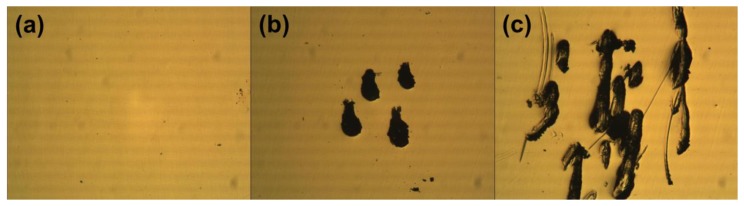
(**a**) An optical microscopic image of an as-prepared flat gold electrode; (**b**) Optical microscope image of a flat gold electrode after five successive measurements using a needle-like probe; (**c**) Optical microscope image of a flat gold electrode after five successive measurements using an alligator clip.

We investigated the optical microscope images of the gold electrodes on a COC plate after probing by conventional methods. The optical microscope images in Figure 1 show how the gold electrodes were damaged or deformed by probing. The flat gold electrode before probing (Figure 1a) was deformed by scratches while probing with conventional probing methods such as a needle-like probe and alligator clip (Figure 1b,c). The mechanically controlled contact of needle-like probes left short scratches, from sliding on the surface of the gold electrode. Manual clipping of the alligator clip left more intense scratches on the gold electrode as the manipulation was subject to shaking and movement. Because scratches are the typical results of mechanical abrasion on metal layers by shear forces, it is deduced that a probing method with a minimal shear force will reduce the mechanical deformation of the gold electrode.

**Figure 2.**
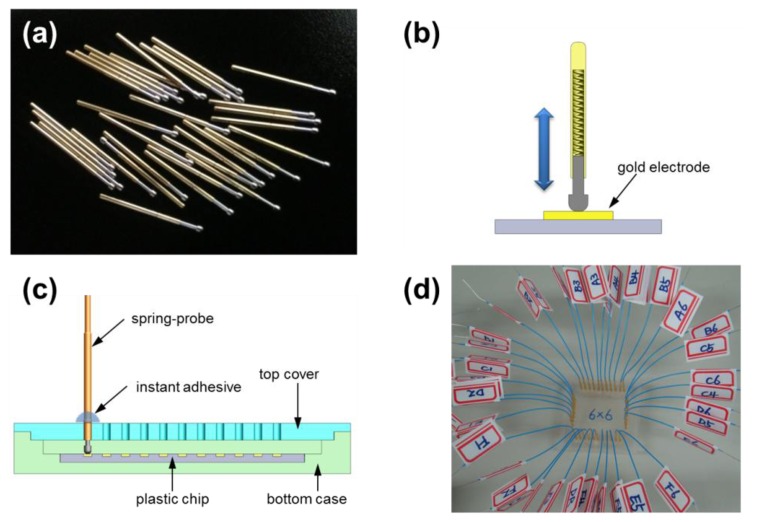
(**a**) Photograph of spring-probes; (**b**) Sectioned drawing of a spring-probe on a gold electrode; (**c**) Sectioned drawing of the spring-probe system; (**d**) Photograph of the fabricated spring-probe system for 24 electrodes.

One possible method to probe an electrode without shear force is to contact and press it vertically. To realize this, a novel probing system was fabricated using spring-probes (Figure 2a), which provided the moderate contact pressure to reduce unstable contacting and deformation of the gold electrode. As a small spring was inserted inside the spring-probe, the vertical restoration force was controlled by the retraction distance of the spring while contacting on the gold electrode (Figure 2b). The new probing system was composed of a top cover and bottom case in which the LOC was placed (Figure 2c). Many holes were formed through the top cover in order to hold spring-probes which were 16.35 mm long and 0.68 mm thick. The spring-probes were firmly attached at right angles to the top cover by an instant adhesive to eliminate shear force. The depth inside the bottom case was precisely adjusted so that spring-probes were half retracted when the top cover was fully closed with a chip inserted in the bottom case. At this moment, the top cover underwent a repulsive force because all the spring-probes produced a restoring force. Figure 2d shows an example of a spring-probe system which had six spring-probes on each side of the top cover. The back end of a single spring-probe was connected to a wire that led to a measuring instrument.

**Figure 3.**
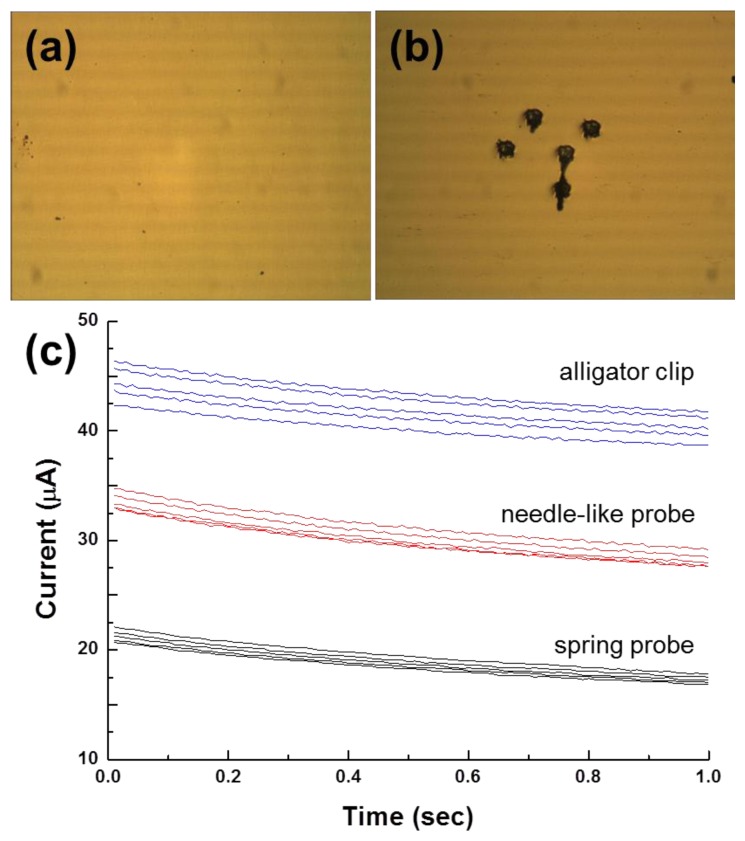
(**a**) Optical microscope image of a flat gold electrode as prepared; (**b**) Optical microscope image of a flat gold electrode after five successive measurements using the spring-probe system; (**c**) Amperometric I-T curves of five successive measurements using the alligator clip, needle-like probe and spring-probe system, respectively. For the comparison, I-T curve data for the needle-like probe and alligator clip were shifted by 10 and 25 μA, respectively.

The probing reliability of the spring-probe system in Figure 2d was compared with those of the needle-like probe and alligator clip. Impact craters of the spring-probe after five successive measurements looked round and similar to each other (Figure 3b). The round shape of the craters meant that the shear force was eliminated well. In addition, the same size of the craters implied that probing of the gold electrode was stable and consistent during the course of contact and withdrawal. Consequently, we expected that signal-to-noise of probing to be improved significantly.

Using this spring-probe system, the reliability of the electrochemical measurements was tested, in contrast to conventional probing methods (Figure 3c). When 5 mV was applied to the working electrode, the current was sent in tens of microamperez. Amperometric I-T curves of the spring-probe system were close to each other, with negligible signal noise. In the case of the needle-like probe, the differences between I-T curves looked similar to those of the spring-probe system but a small signal noise was observed. The curves of the alligator clip were not overlapped but spread. Moreover, signal noise obviously became non-negligible. This result matched well with the observation in Figure 1, as expected. Therefore, the shear-force-free spring-probe system was verified to improve the reliability of electrochemical measurements.

The improvement of electrochemical measurement was evaluated by comparison of the divergence of amperometric I-T curves. For convenience of calculation, we assumed that the integrated current data of each I-T curve is a nominal value (N. V.) that stands for the current level of each curve. Five nominal values were normalized using their average to get rid of the effect of electrode size. Standard deviations of normalized nominal value (N. N. V.) for the spring-probe, needle-like probe and alligator clip were 0.021, 0.031 and 0.072, respectively (Table 1). The divergence in case of the spring-probe was only 30% of that in the case of the alligator clip. This supported the fact that the spring-probe system reduced remarkably the differences in currents between successive measurements. This result was coincided well with what we discussed above.

The spring-probe system in Figure 2d had a drawback in that the position of the spring-probe was fixed at the arranged place. Thus, it was only fit for the specific LOC design which had no more than 24 electrodes in the relevant positions matching the positions of the spring-probes. In research groups or laboratories, various LOC designs are evaluated, so if one must fabricate a spring-probe system per each single design, it would be wasteful of time and effort.

**Figure 4.**
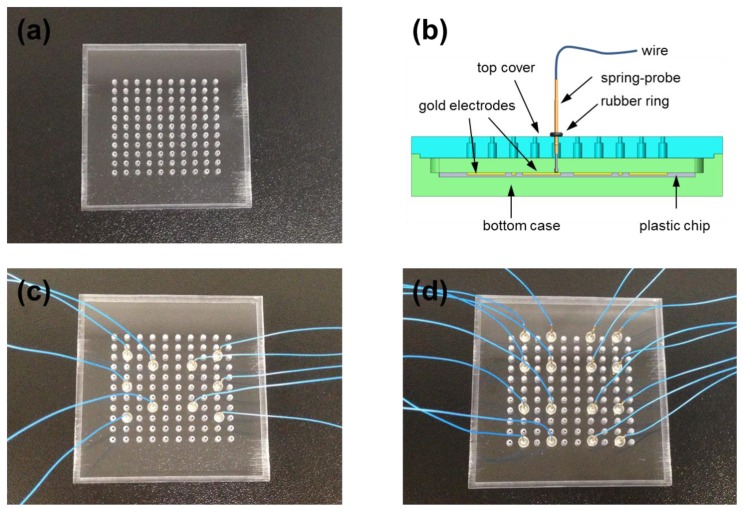
(**a**) Photograph of the top cover of a universal spring-probe system; (**b**) Sectioned drawing of the universal spring-probe system; (**c**) Photograph of an example of a universal spring-probe system for 10 electrodes; (**d**) Photograph of an example of a universal spring-probe system for 16 electrodes.

To enhance the applicability of the spring-probe system, a universal spring-probe system was designed next, as shown in Figure 4. The top cover of the universal spring-probe system had equally-spaced holes to accommodate any position of electrodes according to the design of LOC under testing (Figure 4a). When spring-probes were inserted upwards into the holes from below, the knot in the middle of the spring-probe got stuck to the narrow part of the hole. At this time, if a rubber ring was put in from the wire end, the spring-probe was tightly connected to the top cover without using any instant adhesive (Figure 4b). The rubber ring was small enough to provide the proper friction, preventing the spring-probe from falling out of the top cover.

Examples of this reconfigurable assembly are shown in Figure 4c,d. Spring-probes in a configuration for 10 electrodes (Figure 4c) were easily relocated to the positions for needed for 16 electrodes (Figure 4c) within minutes. For this reason, the universal spring-probe system was expected to meet various LOC designs.

### 3.2. Detection of DNA Hybridization Using the Universal Spring-Probe System

**Figure 5.**
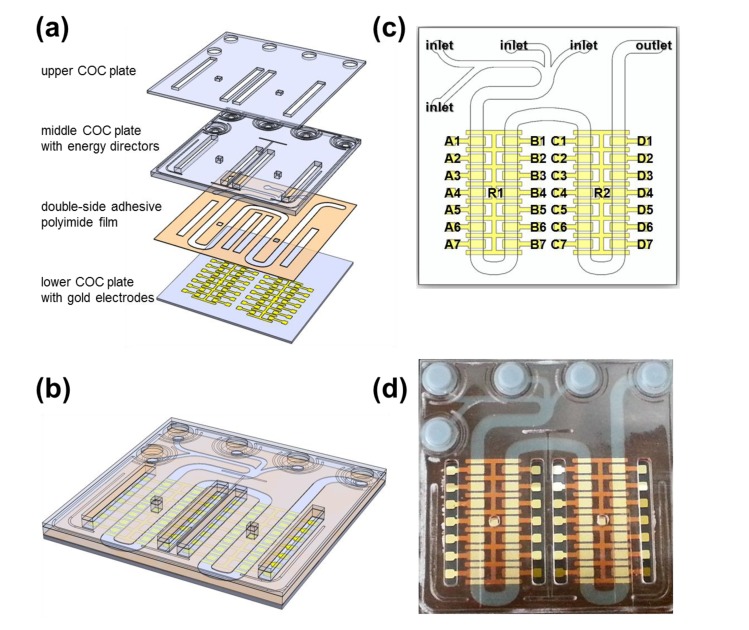
(**a**) Drawing of the components of a LOC designed for electrochemical detection of DNA hybridization; (**b**) Drawing of the assembled microfluidic lab-on-a-chip; (**c**) Layout of the LOC that included four inlets, one outlet and 30 gold electrodes; A1, A3, A5, A7, B1, B3, B5, B7, C1, C3, C5, C7, D1, D2, D5 and D7 were assigned to the working electrodes. A2, A6, B2, B6, C2, C6, D2 and D6 were assigned to the counter electrodes. R1 and R2 were assigned to the reference electrodes. A4, B4, C4 and D4 were not assigned; (**d**) Photograph of the fabricated LOC.

The application of the universal spring-probe system was demonstrated by electrochemical detection of DNA hybridization. Three COC plates were designed to make a LOC with open areas, exposing gold electrodes for universal spring-probe system (Figure 5a,b). Double-side adhesive polyimide film bonded the lower and middle COC plate together, leaving a microfluidic channel and opening for probing. The microfluidic channel started from four inlets in the top left corner of the LOC, ran through 30 gold electrodes and then ended at the outlet in the top right corner (Figure 5c). All gold electrodes were named and then divided into sets designated as A, B, C and D for discrimination.

**Figure 6.**
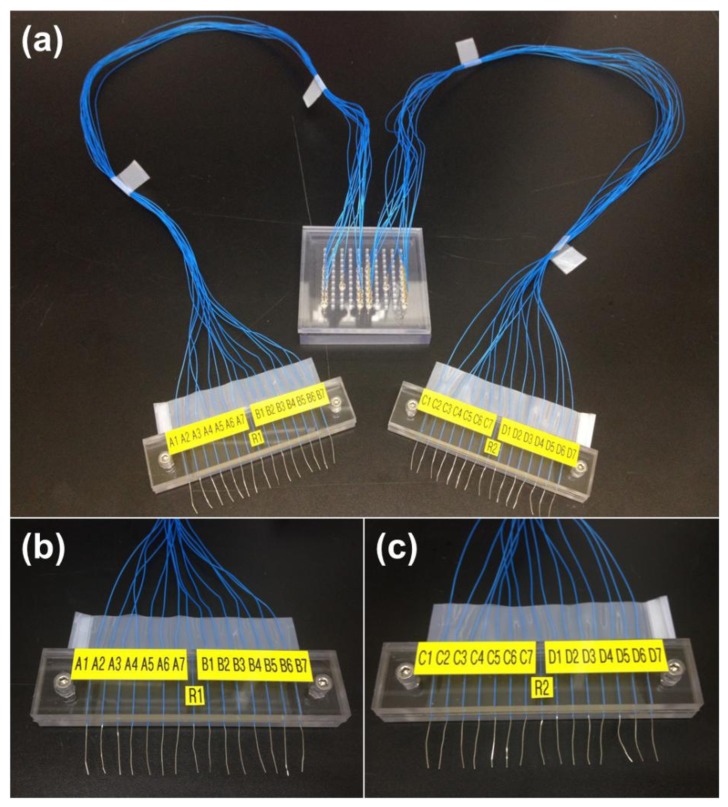
(**a**) Photograph of the universal spring-probe system designed for electrochemical detection of DNA hybridization; (**b**) Close-up photograph of the labeled lead wires from the left-side electrodes; (**c**) Close-up photograph of the labeled lead wires from the right-side electrodes.

A universal spring-probe system was constructed to perform electrochemical detection of DNA hybridization using the LOC (Figure 6). Thirty spring-probes were positioned on the universal top cover at the right place where the corresponding gold electrodes were just below. Lead wires were labeled to avoid confusion and then arranged into two groups.

Probe DNA for alligator clipping was immobilized at the working electrodes of sets A and D, while probe DNA for the universal spring-probe system was placed at the working electrodes of the B and C sets. Cyclic voltammograms of the working electrodes were acquired before and after the hybridization process. Using the universal spring-probe system, two sets of 10 measurements, for a total of 20 measurements, were easily conducted in a minimum time. However, another 20 measurements for alligator clipping demanded significant attention not to cause big scratches, taking a longer time than with the universal spring-probe system.

**Figure 7.**
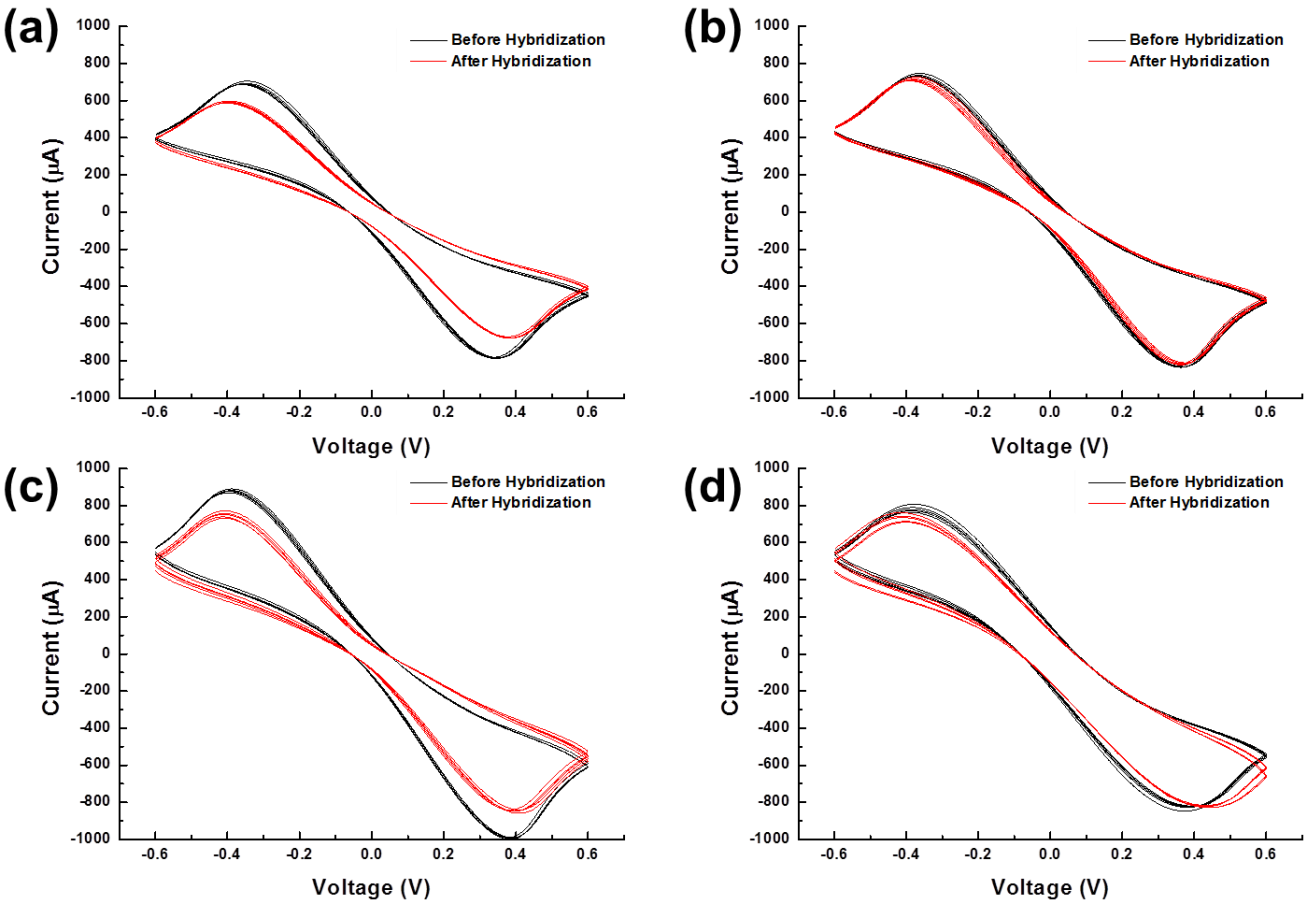
(**a**) C-V voltammograms using the universal spring-probe system before and after hybridization in the case of PM; (**b**) C-V voltammograms using the universal spring-probe system before and after hybridization in the case of MM; (**c**) C-V voltammograms using alligator clips before and after hybridization in the case of PM; (**d**) C-V voltammograms using alligator clips before and after hybridization in the case of MM.

A total of forty voltammograms were plotted in Figure 7 according to the experimental conditions. Oxidation and reduction peaks were significantly reduced after hybridization in the cases of PM (Figure 7a,c). On the other hand, voltammograms of MM were not changed much after hybridization (Figure 7b,d). This result proved that DNA hybridization was successfully detected by both probing methods. However, the voltammograms recorded using the universal spring-probe system were observed to be different from the voltammograms obtained using alligator clips. Each set of five voltammograms for the spring-probe overlapped closer than those of alligator clipping. This observation intuitively indicated that the universal spring-probe system provided more reliable probing than alligator clipping.

**Figure 8.**
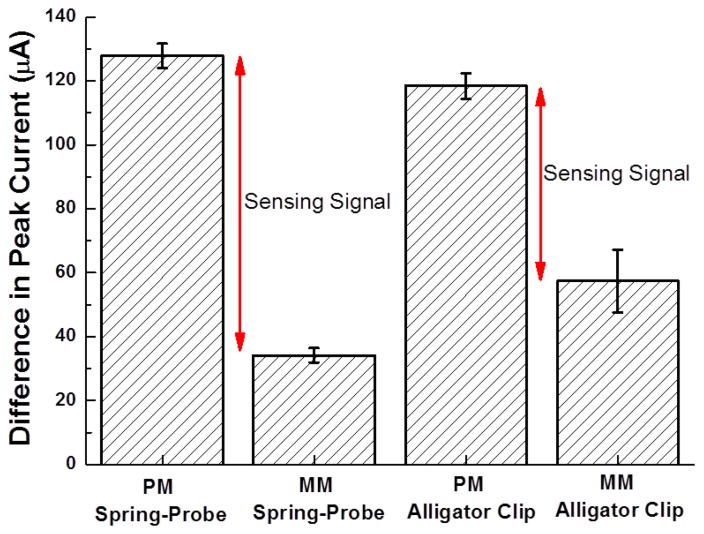
Comparison of the difference in peak currents of C-V voltammograms before and after hybridization using spring-probe and alligator clip, respectively.

The reliability of each probing type was statistically evaluated by the calculation of the difference in peak currents (Figure 8).

The standard error of the difference in peak currents for PM and MM using the spring-probe were 3.8 μA and 2.3 μA, respectively. Meanwhile, the standard error for PM and MM using the alligator clip were 3.9 μA and 9.7 μA, respectively. This result was in accord with the result of amperometric I-T curves in Figure 3c. Furthermore, the gap of the difference in peak currents between PM and MM for the spring-probe sensing of the signal of hybridization, was wider than the gap for the alligator clip. It was deduced that reliable probing eventually enhanced the electrochemical detection sensing signal.

## 4. Conclusions

We have developed a novel system using a spring-probe and the excellence of the spring-probe system was successfully demonstrated in a test of successive electrochemical measurements. The shear-force-free configuration of the spring-probe system minimized the scratches on a gold electrode caused by probe impact, resulting in the most reliable data collection. The universal spring-probe system expanded the adoptability of the spring-probe to any electrode layout. We believe that the universal spring-probe system provides an accurate, efficient and robust method with ease and convenience for electrochemical detection based on LOCs.

## Figures and Tables

**Table 1.** Statistics of nominal values from five successive amperometric measurements.

**Probing Method**		**1st Measurement**	**2nd Measurement**	**3rd Measurement**	**4th Measurement**	**5th Measurement**	**Average**	**Standard Deviation**
**Spring-probe**	N. V.	1956.3	1916.7	1886.9	1861.4	1842.4	1892.7	40.4
	N. N. V.	1.0336	1.0127	0.9969	0.9834	0.9734	1.0000	0.021

**Needle-like probe**	N. V.	2140.9	2074.8	2010.6	1982.0	1970.5	2035.8	63.8
	N. N. V.	1.0516	1.0192	0.9876	0.9736	0.9679	1.0000	0.031

**Alligator clip**	N. V.	1858.3	1802.1	1689.9	1517.7	1623.1	1698.2	122.2
	N. N. V.	1.0943	1.0612	0.9951	0.8937	0.9558	1.0000	0.072

N. V. is nominal value and N. N. V. is normalized nominal value.
